# Review of the Post-IR IRSL Dating Protocols of K-Feldspar

**DOI:** 10.3390/mps3010007

**Published:** 2020-01-14

**Authors:** Junjie Zhang, Sheng-Hua Li

**Affiliations:** Department of Earth Sciences, The University of Hong Kong, Hong Kong 999077, China; junjie@connect.hku.hk

**Keywords:** OSL dating, K-feldspar, post-IR IRSL, standard growth curve (SGC), single-grain

## Abstract

Compared to quartz, the infrared stimulated luminescence (IRSL) of K-feldspar saturates at higher dose, which has great potential for extending the dating limit. However, dating applications with K-feldspar has been hampered due to anomalous fading of the IRSL signal. The post-IR IRSL (pIRIR) signal of K-feldspar stimulated at a higher temperature after a prior low-temperature IR stimulation has significantly lower fading rate. Different dating protocols have been proposed with the pIRIR signals and successful dating applications have been made. In this study, we review the development of various pIRIR dating protocols, and compare their performance in estimating the equivalent dose (D_e_). Standard growth curves (SGCs) of the pIRIR signals of K-feldspar are introduced. Single-grain K-feldspar pIRIR dating is presented and the existing problems are discussed.

## 1. Introduction

Optically stimulated luminescence (OSL) dating is applied to date the last exposure event of a mineral grain to sunlight [1]. The OSL ages are obtained by the equation: Age = D_e_/D_r_, where D_e_ is the equivalent dose, representing the radiation dose that mineral grains have received after being blocked from sunlight, and the D_r_ is the environmental dose rate mainly dominated by the radioactive elements U, Th, K. Quartz has long been used as the ideal mineral for OSL dating, because of its abundance in nature, its signal stability, and its susceptibility to bleaching, especially after the single-aliquot regenerative-dose (SAR) protocol was established [2,3,4]. However, the OSL signal of quartz saturates with relatively low radiation dose, which has limited its application in dating older samples. Studies have shown that the quartz OSL ages were underestimated when the samples were older than ~100 ka [5,6,7], a situation usually attributed to the early saturation behavior of quartz OSL signal [8]. In some case, such as in the aeolian deposits from the Chinese Loess Plateau, age underestimation began at ~70 ka [9,10], which may be related to abnormally short lifetime of the OSL signal [11,12].

Compared to quartz, the infrared stimulated luminescence (IRSL) of K-feldspar has several advantages: the IRSL signal of K-feldspar saturates at higher dose, which has the potential to date older samples; the IRSL signal intensity of K-feldspar is much greater than quartz OSL signal, providing higher data precision; and the K element within K-feldspar grains provides an internal component for the dose rate, which is not affected by variations of the external environment [13,14,15]. However, the application of K-feldspar IRSL dating has long been hampered by a phenomenon called anomalous fading [16,17,18]. The thermally stable luminescence signal of K-feldspar fades under ambient temperature, which is suggested to be a result of quantum tunneling between the electron traps and holes [16,17,18,19]. Fading correction method has been proposed to address this problem [14,20,21]. With this solution, the fading rate of IRSL signal for each individual sample needs to be measured, which requires a large amount of laboratory work. In addition, fading correction for older samples would become more complicated because of the dose-dependent behavior of fading rate [21,22,23,24], and age overestimation after fading correction has been widely reported [25,26,27,28,29]. An isochron dating method has also been proposed [30,31]. In this method, it is assumed that the IRSL signal induced by internal dose does not fade, and D_e_ values of K-feldspar fractions with different grain sizes need to be measured, which is also laborious.

It would be highly advantageous if a non-fading signal of K-feldspar could be identified. Studies that aimed to isolate the non-fading IRSL signals have driven the development of post-IR IRSL (pIRIR) dating protocols, and a detailed review has been published in 2014 [32]. Here, we briefly introduce the pIRIR developments before 2014, and focus on the improvements since 2014.

## 2. Development of pIRIR Dating Protocols

### 2.1. Two-Step IR Stimulation

Thomsen et al. [33] found that the IRSL signal stimulated at 225 °C after a preceding IR stimulation at 50 °C (post-IR IRSL) faded much more slowly. The 50 °C IR stimulation (IR_50_) is supposed to remove the close trap-hole pairs which are prone to tunneling; while the left distant trap-hole pairs stimulated at the subsequent high temperature experience less fading [33,34,35,36]. Following the observation of Thomsen et al. [33], Buylaert et al. [37] tested the two-step post-IR IRSL (pIRIR_50, 225_) dating protocol (Table 1) on K-feldspar extracts from various depositional environments. The fading rate (g_2days_ value) of the pIRIR_50, 225_ signal (mean value, 1.62 ± 0.06%/decade) was significantly lower than the fading rate of the IR_50_ signal (mean value, 3.23 ± 0.13%/decade) [37]. After fading correction, the pIRIR_50, 225_ and IR_50_ signals had similar ages; however, the smaller fading rate made the pIRIR_50, 225_ ages less dependent on the assumptions behind the fading correction model [37].

In order to isolate a more stable pIRIR signal, Thiel et al. [38] increased the temperature of the second-step stimulation from 225 °C to 290 °C (Table 1). The measured fading rate of the pIRIR_50, 290_ signal of loess samples from Austria was 1.0–1.5%/decade, compared to 2.1–3.6%/decade of the IR_50_ signal [38]. However, based on observations that the natural pIRIR_50, 290_ signal of a sample from below the Brunhes/Matuyama geomagnetic reversal boundary was saturated and that a quartz sample also showed a measured fading rate of 1.3 ± 0.3%/decade, Thiel et al. [38] suggested that the fading rate of the pIRIR_50, 290_ signal was a laboratory artifact, and fading uncorrected pIRIR_50, 290_ ages were the most appropriate. Buylaert et al. [39] systematically tested the pIRIR_50, 290_ protocol and concluded that it was a robust dating method for Middle and Late Pleistocene sediments without fading correction. 

The pIRIR signals stimulated at higher temperatures (e.g., pIRIR_50, 225_, pIRIR_50, 290_, pIRIR_200, 290_) are more stable than the IR_50_ signal; however, they are also more difficult to bleach [33,37,38]. Thus, the residual doses of the high-temperature pIRIR signals are a concern in dating. Buylaert et al. [40] reported that the residual doses of the pIRIR_50, 225_ and pIRIR_50, 290_ signals from modern aeolian samples of the Chinese Loess Plateau were in the range of 2–8 Gy and 5–19 Gy, respectively. The pIRIR_50, 290_ residual doses of three modern samples from an alluvial bar in Murray et al. [41] were measured to be 5–10 Gy. While quite high pIRIR_50, 290_ residual doses (20–55 Gy) were reported for modern glaciofluvial sediments from a glaciated bay [42]. In various solar bleaching experiments, the measured residual doses of the pIRIR signals varied within a wide range, from less than 2 Gy to >40 Gy [38,39,43,44,45], and the residual doses increased systematically with higher preheat temperature and stimulation temperature [46,47,48]. A positive relationship between the residual doses and D_e_ values has been documented in numerous studies [39,49,50,51,52,53,54]. With a fixed bleaching time, a linear function between the residual doses and D_e_ values can be determined, and a minimal residual dose can be obtained by extrapolating to D_e_ = 0 Gy. The minimal residual dose of the pIRIR_50, 225/290_ signal is mostly in the range of 4–7 Gy, which represents the contribution of an un-bleachable component that ubiquitously exists in fully-bleached samples [39,49,50,52,53,54]. In addition, some studies showed that with extremely long bleaching durations, the residual doses were also reduced to <10 Gy [29,51,52]. In summary, the residual doses of pIRIR_290_ signal for partially-bleached sediments (e.g., glaciofluvial deposits) could be high (up to ~50 Gy), but the residual doses for well-bleached sediments are, in most cases, smaller than 10 Gy. Some studies argued that bleaching under different natural conditions (e.g., sub-aqueous and sub-aerial) had different efficiency due to different spectrum, which questioned the strategy of residual dose subtraction for D_e_ measurements [51,55].

For well-bleached old samples, a D_e_ overestimation of <10 Gy would result in an age overestimation of <~3 ka (e.g., for typical loess sediments), which has small effect. However, it becomes significant for young samples (e.g., of Holocene ages). In order to reduce the effect of residual dose, Reimann et al. [27] proposed a modified two-step pIRIR protocol with the first IR stimulation at 50 °C and the second IR stimulation at 180 °C, together with a preheat temperature of 200 °C (Table 1). The residual dose of the pIRIR_50, 180_ signal was reduced to ~1 Gy [27]. Reimann et al. [27] showed that the pIRIR_50, 180_ signal was sufficiently stable to date well-bleached middle Holocene and late Pleistocene samples, as the pIRIR_50, 180_ ages agreed very well with quartz OSL ages and radiocarbon ages. To make it suitable to date even younger samples (e.g., of hundreds of years), the second IR stimulation was decreased to 150 °C, with a preheat treatment at 180 °C [28,56]. In several studies, the second IR stimulation temperature was changed to 170 °C with the preheat temperature at 200 °C to date Holocene aeolian deposits [57,58,59].

### 2.2. Multi-Step IR Stimulation

**Figure 1:**
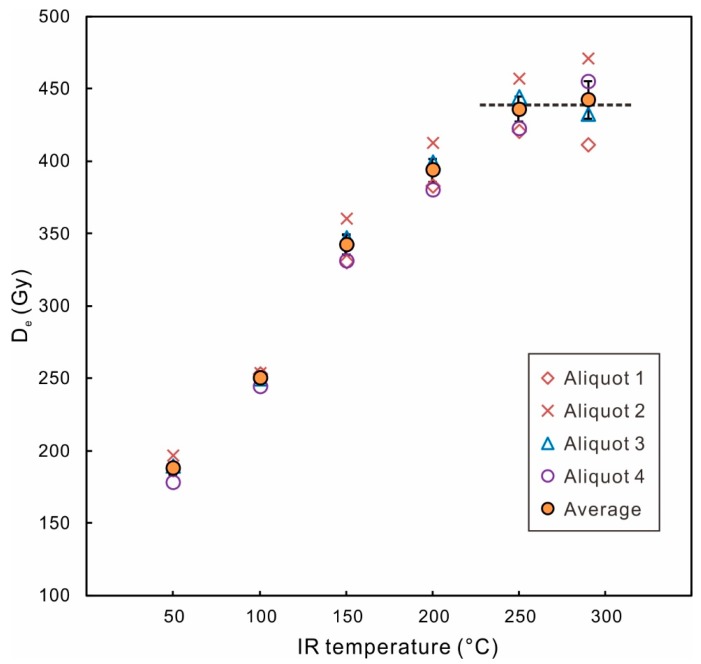
D_e_ versus IR stimulation temperature in the MET-pIRIR protocol. The sample used is 14LC-11.0, from the top of the L2 layer (second loess layer, corresponding to Marine Isotope Stage 6) in the Luochuan section, Chinese Loess Plateau (Zhang et al. [72]). Four aliquots were measured. The error bar of the average is the standard error calculated from four aliquots.

Li and Li [26] proposed a multiple-elevated-temperature (MET) pIRIR dating protocol, with five-step IR stimulations from 50 °C to 250 °C with an increment of 50 °C (Table 1). An advantage of this protocol is that there are multiple D_e_ values corresponding to IR stimulations at different temperatures. Once the D_e_ reaches a plateau in the high temperature range, it would provide firm evidence that the high-temperature pIRIR signals are sufficiently stable and experience negligible anomalous fading (Figure 1) [26]. Later, the five-step MET-pIRIR protocol was modified to a six-step MET-pIRIR protocol, with the highest IR stimulation temperature increased from 250 °C to 300 °C, and the preheat temperature increased from 300 °C to 320 °C [63]. For the studied loess and desert samples from north China, the fading rates of the IR_50_ signal were 3–5%/decade, while the fading rates of MET-pIRIR_250_ or MET-pIRIR_300_ signals were very close to zero [26,63].

From the solar bleaching experiments, residual doses of the MET-pIRIR_250_ signal were mostly in the range of 2–10 Gy, although in some cases they were up to ~20 Gy [26,64,65,66,67,68]. In order to make the MET-pIRIR protocol suitable for dating Holocene sediments, Fu and Li. [69] developed a modified low-temperature MET-pIRIR protocol. The preheat temperature was set to 200 °C and the five-step IR stimulation was performed from 50 °C to 170 °C with a step of 30 °C. The residual dose of the MET-pIRIR_170_ signal was generally less than 1 Gy [69]. For Holocene samples, Fu and Li. [69] observed an age plateau between the stimulation temperatures of 110–170 °C. The five-step stimulation can be simplified to a three-step stimulation at temperatures of 110, 140, 170 °C. With the three-step protocol, the age plateau still existed at the stimulation temperatures of 140 °C and 170 °C, indicating that the pIRIR signals at 140 °C and 170 °C can be considered as sufficiently stable over the timescale of Holocene period [69]. Thus, Fu and Li. [69] further simplified the three-step protocol to a two-step protocol, with IR stimulations at 110 °C and 170 °C. Although no age plateau can be observed with the two-step IR stimulation, the pIRIR_110, 170_ signal can still provide identical ages as the five-step pIRIR_170_ signal, the three-step pIRIR_170_ signal, and the quartz OSL signal. However, when the first IR stimulation temperature was decreased from 110 °C to 50 °C, the corresponded pIRIR_50, 170_ ages were slightly underestimated [69].

As pIRIR signals stimulated at higher temperatures are more difficult to be bleached, several studies have proposed that the different bleaching rates of MET-pIRIR signals can be applied to infer the degree of bleaching, and thus to trace the transport history of sediments [70,71].

### 2.3. Modified Protocols to Extend the Dating Limit

The two-step pIRIR or MET-pIRIR protocols discussed above are all based on the conventional SAR protocol, with a subsequent test dose signal (T_x_) to correct for the change in the sensitivity of the regenerative dose signal (L_x_). Each aliquot is measured repeatedly with several cycles to build its individual growth curve [8]. The growth curve of the luminescence signal with the radiation dose can be fitted with a single saturating exponential function:

(1) $$I=I_{0}+I_{max}*\left(1-e^{-D/D_{0}}\right),$$

where *I* is the luminescence signal, *D* is the radiation dose, and *D*_0_ is called the characteristic saturation dose which quantifies the saturation behavior of the signal. 

For quartz, D_0_ varies significantly between different grains and samples, but in most cases it is smaller than 200 Gy [73,74,75,76]. It is suggested that for reliable dating with quartz, the D_e_ should not exceed the 2D_0_ limit [8]. Although D_0_ of the K-feldspar pIRIR signal varies with different samples and experimental parameters (e.g., test dose, IR stimulation temperature), it is usually around 200–500 Gy with the conventional SAR protocol (Table 2). Applying the 2D_0_ limit, the dating range of the K-feldspar pIRIR signals would not exceed 1000 Gy. To extend the dating limit, modified protocols have been proposed based on the dose-dependent sensitivity of the MET-pIRIR signals from K-feldspar [61,62,77]. Li et al. [62] applied the multiple-aliquot regenerative-dose (MAR) protocol, but added a second test dose (T_2_) after a ‘cutheat to 600 °C’ treatment behind the first test dose (T_1_). The T_1_/T_2_ signal was applied to represent the dose-dependent sensitivity. For the MET-pIRIR_250_ signal, the D_0_ of the T_1_/T_2_ signal was ~740 Gy, which was significantly larger than that of the L_x_/T_1_ signal (~340 Gy). In addition, the L_x_/T_2_ signal also had a D_0_ of ~770 Gy, close to that of the T_1_/T_2_ signal. Chen et al. [77] presented that with the L_x_/T_2_ signal, the D_e_ of a sample from the fifth paleosol layer (S5) of the Chinese loess sequence, corresponding to Marine Isotope Stage (MIS) 13–15, was estimated to be 1360 ± 200 Gy, which was broadly consistent with the expected D_e_ of 1550 ± 72 Gy. The modified MAR protocol is termed ‘MAR with heat’ in the text below (Table 1). Li et al. [61] modified the conventional SAR protocol, by adding a solar beaching treatment behind each cycle—‘SAR with solar’ (Table 1). It was found that solar bleaching was able to reset the luminescence sensitivity. Hence, the regenerative dose signal (L_x_) or the test dose signal (T_x_) can be used alone to estimate the D_e_. Here, the T_x_ is used to represent the dose-dependent sensitivity. While the D_0_ of the L_x_/T_x_ signal was ~400 Gy, the D_0_ of both the L_x_ and T_x_ signals was ~800 Gy [61].

**Figure 2:**
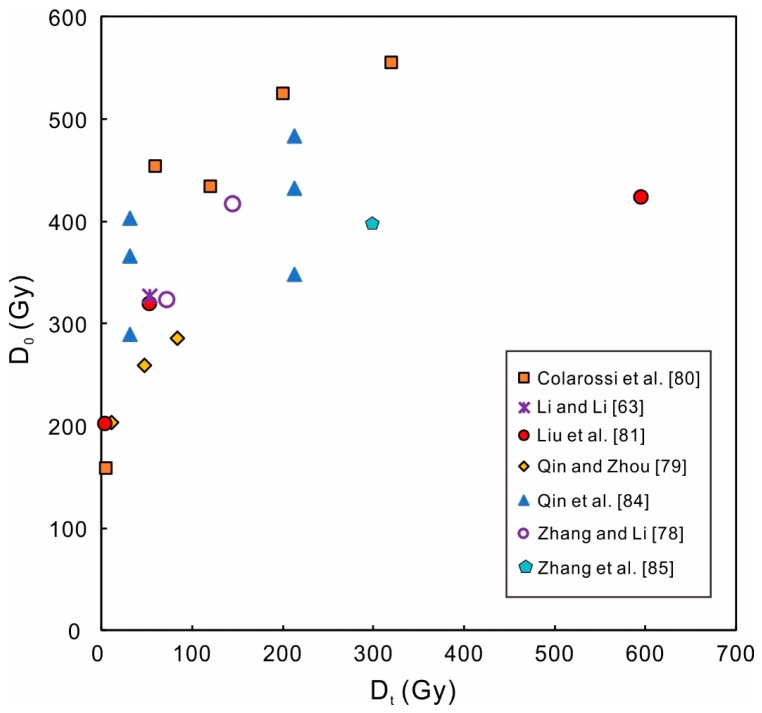
Characteristic saturation dose (D_0_) of the pIRIR signals versus the test dose (D_t_), with the SAR protocol. Data are from Table 2. A general positive relationship exists between D_0_ and D_t_. Please note that the pIRIR signals in this graph includes different kinds of signals, such as pIRIR_50, 170_, pIRIR_50, 225_, pIRIR_50, 290_, MET-pIRIR_250/300_ signals. So the D_0_ is scattered even with the same D_t_.

These modified MAR and SAR protocols have greatly increased the D_0_ of the K-feldspar pIRIR signal, and have extended the dating limit of K-feldspar to ~1500 Gy. Zhang and Li [78] proposed that a D_0_ of ~800 Gy was very likely to be the intrinsic property of the pIRIR signals. In the conventional SAR protocols, the signals of the test dose (D_t_) would be overestimated due to the effect of the preceding regenerative dose [43,78,79,80,81,82]. Multiple hypotheses have been proposed to account for the overestimation, such as the thermally transferred signal [43,79], signal inheritance [80,81], and dose dependent sensitivity change [62,82]. The test dose signal following a larger regenerative dose would be overestimated in a higher degree, and the corresponding L_x_/T_x_ would be underestimated more significantly; hence the fitted growth curves have apparently lower D_0_ values (200–500 Gy) compared to the intrinsic D_0_ (~800 Gy). A larger test dose would reduce such an effect, thus a positive relationship between D_0_ and D_t_ has been observed in numerous studies (Figure 2) [78,79,80,81,83,84].

## 3. Comparison between Different pIRIR Protocols

Several studies have been performed to investigate the pIRIR_290_ D_e_ dependence on the prior-IR stimulation temperature, and these studies suggested that the D_e_ values did not change significantly with the prior-IR stimulation temperature varied in the range of 50–260 °C [39,52,53,54,86]. From a comparison of the D_e_ values obtained with the pIRIR_50, 290_, pIRIR_200, 290_, MET-pIRIR_250_ signals, Li and Li [60] showed that the estimated D_e_ values were consistent between the three signals when D_e_ was less than ~400 Gy; however, when the expected D_e_ exceeded ~400 Gy, the pIRIR_50, 290_ signal had underestimated D_e_ results compared to the other two signals. Li and Li [60] suggested that the 50 °C prior-IR stimulation was too weak to completely remove the prone-to-fade signal. Qiu and Zhou. [87] compared the performance of four signals, which were pIRIR_50, 290_, pIRIR_200, 290_, three-step pIRIR_200, 290_ signal with first stimulation at 50 °C, second stimulation at 200 °C and last stimulation at 290 °C, IRoff-pIRIR_200, 290_ signal with isothermal holding (IR-off) for 200 s before the 290 °C IR stimulation, respectively. The D_e_ values of their tested samples were within the range of 400–900 Gy. Only the pIRIR_50, 290_ signal had underestimated the D_e_ values, whereas the other three signals provided consistent D_e_ results [87]. Buylaert et al. [39] revealed that when the prior-IR stimulation temperature was increased from 50 °C to 200 °C, the intensity of corresponded pIRIR_200, 290_ signal was only 7% of the pIRIR_50, 290_ signal. However, it was later found that the pIRIR_290_ signal intensity was still sufficiently high to guarantee precise measurements even when the prior-IR stimulation temperature was as high as 260 °C, and thus Buylaert et al. [88] applied the pIRIR_200, 290_ protocol to date the last interglacial paleosol (S1) in the Chinese Loess Plateau. Stevens et al. [89] reported that D_e_ values were underestimated when the prior-IR stimulation temperature was below 140 °C, but a D_e_ plateau had been reached when the prior-IR stimulation temperature was ≥170 °C; and the pIRIR_200, 290_ protocol was adopted to date the Chinese loess back to the S2 layer (second paleosol layer, corresponding to MIS 7). Ito et al. [29] carried out prior-IR stimulation temperature test on marine terrace deposits from Japan, and the results showed that the pIRIR_290_ D_e_ plateau existed when the prior-IR temperature was within the range of 100–200 °C; while the pIRIR_290_ D_e_ was underestimated with a prior-IR temperature of 50 °C and overestimated with a prior-IR temperature of 250 °C. A study performed on rock slices showed that the pIRIR_290_ signal from naturally saturated slices was close to the laboratory saturation level only when the first-IR stimulation temperature was high (e.g., 200 °C or 250 °C) [81]. These studies suggest that the first-IR stimulation is better to be performed at a higher temperature (e.g., 200 °C) when dating older samples with the pIRIR_290_ signal.

All of the foregoing comparisons in this section are based on the SAR protocol. However, the upper dating limit of these pIRIR SAR protocols has seldom been studied. A sample from the fifth loess layer (L5) of the Mangshan loess-palaeosol sequence of China was dated to be 401 ± 35 ka with the pIRIR_200, 290_ protocol, which was much younger than the expected age within MIS 12 [87]. Zhang et al. [85] showed that with the SAR protocols, irrespective of the pIRIR_200, 290_ signal or the MET-pIRIR_250/300_ signal, the D_e_ values were underestimated when the expected D_e_ exceeded ~800 Gy, whereas the ‘MAR with heat’ protocol could provide reliable D_e_ estimates. The SAR D_e_ underestimation began to occur at the D_e_ value of ~800 Gy, which was close to twice the D_0_ (~400 Gy with SAR protocol) for samples in Zhang et al. [85]. This indicates that the empirical 2D_0_ limit is also applicable to D_e_ measurements with K-feldspar.

**Figure 3:**
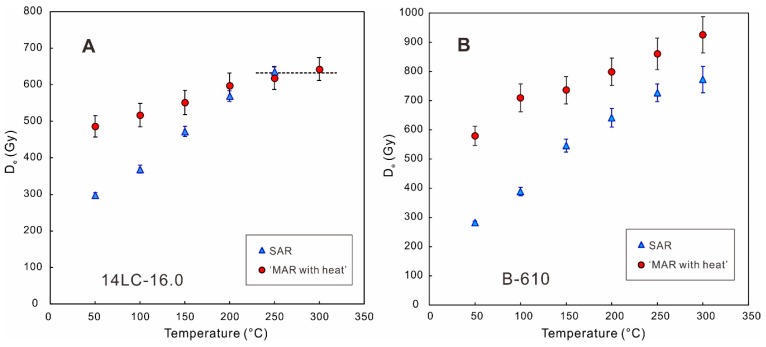
Comparison of D_e_ values obtained by the conventional SAR protocol (Li and Li [63]) and the modified ‘MAR with heat’ protocol (Li et al. [62]), with a six-step IR stimulation from 50 °C to 300 °C. (**A**) Sample 14LC-16.0 is from the base of the L2 layer (second loess layer, MIS 6) in the Luochuan section, Chinese Loess Plateau. The higher degree of SAR D_e_ underestimation compared to MAR D_e_ at low IR stimulation temperatures is due to the failure of sensitivity correction. Note that the D_e_ values are still consistent between the SAR and MAR protocols at higher IR stimulation temperatures (250 °C and 300 °C). (**B**) Sample B-610 is from the Jingbian section of Chinese Loess Plateau. Note that the SAR D_e_ values are still underestimated at high IR stimulation temperatures compared to the MAR D_e_ values, because D_e_ is already larger than 800 Gy. Figure 3B is modified from Zhang et al. [85].

Figure 3A shows that for a loess sample with the D_e_ of ~640 Gy, the SAR D_e_ values are smaller compared to the MAR D_e_ values for the low-temperature signals, but they become similar for the high-temperature (250 °C and 300 °C) signals. Several studies have illustrated that sensitivity correction in the first cycle (natural signal measurement) of the SAR protocol is unsuccessful for the low-temperature IRSL signals (e.g., IR_50_) of K-feldspar when a high preheat temperature (e.g., >200 °C) is applied, and D_e_ underestimation exists for those low-temperature signals [67,84,90,91,92]. Age underestimation of the low-temperature IRSL signals has two sources with the SAR protocol—the anomalous fading and the failure of sensitivity correction. Some studies proposed that the failure of sensitivity correction was related to the increased electron trapping probability caused by the first preheat treatment [67,90,91]; while a recent study suggested that it was a combined effect of the decrease in the electron trapping probability and the increase in recombination probability [84]. The sensitivity correction of the high-temperature pIRIR signals is generally acceptable [67,84,91,92], which is why consistent SAR D_e_ and MAR D_e_ values can be obtained at the temperatures of 250 °C and 300 °C for relatively young samples (Figure 3A). However, the SAR D_e_ value becomes smaller than the MAR D_e_ value for a sample with D_e_ of ~900 Gy (Figure 3B) [85]. Despite of the generally successful sensitivity correction of the high-temperature pIRIR signals, both slight SAR D_e_ underestimation or overestimation can still occur, depending on the stimulation temperature of the pIRIR signals, the size of the D_e_ and the test dose, as well as sample origins [52,67,82,84,89]. Zhang et al. [85] performed dose recovery tests on the samples from their study, and observed a 10% overestimation for the dose recovery ratios with a recovery dose of 900 Gy, which cannot explain the SAR D_e_ underestimation for these old samples.

The empirical 2D_0_ limit was initially proposed for quartz [8]. A recent study argued that the D_e_ underestimation beyond the 2D_0_ limit of quartz was caused by the rejection of the ‘saturated’ aliquots or grains which resulted in a truncated D_e_ distribution [93]. Instead of the conventional ‘mean D_e_’ method, Li et al. [93] proposed a ‘mean L_n_/T_n_’ method to overcome this problem. The ‘mean L_n_/T_n_’ method applies the mean of re-normalized natural signal (L_n_/T_n_) to calculate the final D_e_, thus no grains or aliquots would be abandoned when their natural signals lie above the saturation level of the growth curve. This method has been successfully applied in dating quartz of archeological cave sediments with D_e_ values up to ~300 Gy [94]. However, for the K-feldspar samples whose SAR D_e_ values were underestimated in Zhang et al. [85], no ‘saturated’ aliquots were observed and discarded. Applying the ‘mean L_n_/T_n_’ method cannot overcome the D_e_ underestimation of K-feldspar in Zhang et al. [85]. Several studies revealed that the natural dose response curve of quartz saturated at lower dose than the laboratory dose response curve, and they suggested it was the reason for quartz D_e_ underestimation in the high dose range [74,95,96,97]. Whether the natural dose response curve of K-feldspar also saturates at lower dose than the laboratory dose response curve needs further research.

## 4. Standard Growth Curves

The standard growth curve (SGC) was initially proposed for quartz to simplify the measurement procedure of D_e_ estimation [98]. Test dose standardized OSL signal (L_x_/T_x_ × D_t_) was applied to construct the SGC [98]. With the SGC, only the sensitivity corrected natural signal (L_n_/T_n_) needs to be measured to estimate the D_e_, which has greatly improved the efficiency of OSL dating [98]. However, some studies reported that if different D_t_ values were used, the L_x_/T_x_ × D_t_ signal were still deviated from each other, with larger L_x_/T_x_ × D_t_ for larger D_t_ [99,100]. Later, a regenerative dose normalization (re-normalization) procedure was proposed [101,102]. For each individual aliquot, the sensitivity corrected signals (L_x_/T_x_) are firstly re-normalized by a signal (L_r1_/T_r1_) from the aliquot itself. The re-normalized signal (*I*) is obtained by the following equation:

(2) $$I=\frac{L_{x}/T_{x}}{L_{r1}/T_{r1}},$$

where *L_x_*/*T_x_* are the sensitivity corrected signals of all the regenerative doses and *L_r_*_1_/*T_r_*_1_ is the sensitivity corrected signal of a specific regenerative dose (D_r1_). The *I* values of different aliquots and samples are plotted together, against the regenerative doses, to fit the SGC with appropriate functions such as the single saturating exponential (SSE) function, double saturating exponential (DSE) function, and general-order kinetic (GOK) function [103]. To estimate D_e_, two cycles of the conventional SAR protocol need to be performed. The first cycle is to measure the natural signal (*L_n_*/*T_n_*) and the second cycle to measure the signal (*L_c_*/*T_c_*) of a regenerative dose, which is here termed the calibration dose (D_c_). It is better to use a D_c_ that is close to the expected D_e_ value [78,102,104]. The *L_c_*/*T_c_* is used to calibrate the *L_n_*/*T_n_*:

(3) $$f\left(D_{e}\right)=f\left(D_{c}\right)\frac{L_{n}/T_{n}}{L_{c}/T_{c}},$$

where the *f*(*D_c_*) is the corresponded functional value of *D_c_* on the SGC. The SGC *D_e_* can be estimated from the *f*(*D_e_*) according to the SGC function.

The D_t_ applied in the SGC construction of K-feldspar was 24–66 Gy in Li et al. [102]. Due to the small size of the test dose, no dependence had been observed between the shape of the SGC and the D_t_ [102]. Zhang and Li [78] applied two larger D_t_ (72.5 Gy, 145 Gy) for SGC constructions, and reported that the SGC with a D_t_ of 145 Gy had a larger D_0_ than the SGC with a D_t_ of 72.5 Gy. As illustrated above, D_0_ generally increases with D_t_ (Figure 2). Zhang and Li [78] suggested that the D_t_ used for SGC D_e_ estimation should be close to the D_t_ used for SGC construction. Identical D_t_ for SGC construction and D_e_ estimation would always be the best choice.

A least-squares normalization procedure (‘LS-normalization’) was proposed to establish the SGCs for quartz from Haua Fteah cave, Libya [76]. Later, this LS-normalization procedure has also been applied in constructing SGCs for K-feldspar [92,105]. The ‘LS-normalization’ can further reduce the inter-grain and inter-aliquot variation of individual growth curves. It involves an iterative re-scaling and fitting process [76]. First, a starting curve is chosen. Then, individual growth curve of each aliquot or grain is re-scaled by multiplying a factor to make the sum of squared residuals—the difference between the observed values and the fitted values—is the smallest. All the rescaled-data are fitted again with a certain function. Iterate the re-scaling and fitting process until there is negligible change (<1%) in the results. The procedure can now be easily performed with the lsNORM function in the R package ‘numOSL’ [106].

For quartz, different grains or aliquots have quite different D_0_ values, and different SGCs need to be built for quartz groups with different saturation behaviors [76,94]. In Figure 2, the D_0_ values of K-feldspar are scatted even when the D_t_ is similar. That is because the figure includes different kinds of pIRIR signals (pIRIR_50, 170_, pIRIR_50, 225_, pIRIR_50, 290_, MET-pIRIR_250_, etc.). Also, the maximum regenerative doses used to build the growth curves in different studies are also different. Usually, the D_0_ would increase when the maximum regenerative dose is larger [107]. When using the same D_t_, the identical pIRIR signal from K-feldspar has quite similar saturation behavior between different samples [78,102,105]. Individual growth curves of different K-feldspar grains or aliquots from different continents become very close to each other after re-normalization or re-scaling, which indicates the existence of a global SGC of K-feldspar [102,105].

With a SGC, the machine time needs to estimate the D_e_ is only 1/3 of the time with a standard SAR approach, but the D_e_ values obtained by the SGC method are almost identical to the SAR D_e_ values [78,102]. Similar to quartz, the SGC also provides the ‘mean L_n_/T_n_’ solution to date K-feldspar samples with natural signals close to saturation [108], which cannot be accomplished with the conventional SAR approach.

## 5. Single-Grain Dating

The conventionally used aliquots in OSL dating usually contain hundreds of grains. The D_e_ values determined by aliquots are the mean of multiple grains. For partially-bleached samples, it would result in age overestimation. Single-grain dating measures the D_e_ values of individual grains, thus it has great advantages in dealing with partially-bleached sediments. By applying certain age models (e.g., [109,110,111,112,113]), the portion of fully-bleached grains can be distinguished and the last exposure event can be dated. Single-grain dating is also applicable to sediments that were well-bleached at deposition, but suffered disturbance after burial which resulted in mixing between different-aged grains (e.g., [114,115,116,117,118]). Several studies have applied single-grain dating of K-feldspar with the low-temperature IRSL signal (e.g., at 50 °C) [119,120,121,122,123,124,125]. Different methods were applied to overcome the fading problem, such as the *fadia* method [119,120,121], the isochron method [122], isolating ‘zero’-fading grains [123], and fading correction [124,125]. After the pIRIR dating protocol was established, single-grain pIRIR dating with K-feldspar has been reported in numerous studies [43,108,126,127,128,129,130,131,132,133,134,135,136,137]. The machine time can be saved by performing prior-IR stimulations on all the grains simultaneously [108,129,137].

However, a general trend has been observed that brighter K-feldspar grains (higher signal sensitivity) yield higher D_e_ values [108,126,129,130,137]. Reimann et al. [126] applied pIRIR_50, 180_ dating protocol on K-feldspar single grains from southern Baltic Sea coast, NE Germany. A dependence of D_e_ values on the brightness of individual grains was observed—with higher D_e_ values for brighter grains, and only the brightest 30% of the K-feldspar grains yielded the mean single-grain pIRIR_50, 180_ ages that agreed with the age control [126]. As Reimann et al. [126] found that the fading rates of grains with different brightness were still close to each other, they suggested that the single-grain D_e_ dependence on brightness might be due to the different K contents in the grains (dimmer grains may contain less K). Brown et al. [129] performed K-feldspar single-grain pIRIR_50, 225_ dating for alluvial fan deposits in Baja California Sur, Mexico. In their study, the grains with brighter signal were found to have smaller fading rates. Rhodes [130] also reported a positive relationship between the grain brightness and D_e_ values, when dating individual K-feldspar grains from different locations with the pIRIR_50, 225_ signal. Rhodes [130] proposed an improved separation method to select the K-feldspar grains with density smaller than 2.565 g/cm^3^—the ‘Super-K’ grains with higher brightness, rather than the regular K-feldspar fraction (density < 2.58 g/cm^3^).

Jacobs et al. [108] carried out K-feldspar single-grain pIRIR_200, 275_ dating on sediments of the Denisova Cave in southern Siberia. For more than half of their samples, the weighted mean D_e_ values increased with a higher ’brightness threshold’ of the grains (T_n_), and similar pattern was also observed in the dose recovery ratios [108]. To make accurate D_e_ estimation, Jacobs et al. [108] determined a ‘threshold’ T_n_ for each sample, above which a mean D_e_ ‘plateau’ could be reached. The measured K contents of 60 individual K-feldspar grains (density < 2.58 g/cm^3^) from Jacobs et al. [108] were mostly in the range of 12–14%, and no dependence of brightness on the K contents was observed. Guo et al. [137] compared the single-aliquot and single-grain MET-pIRIR_170_ D_e_ results of K-feldspar samples from the Nihewan Basin, northern China. The mean D_e_ values of single-grain results were smaller than the those of the single-aliquot results (single-aliquot D_e_ was dominated by brighter grains inside the aliquot). Applying the ‘brightness threshold’ method, the mean single-grain D_e_ values corresponding to brighter grains became close to the single-aliquot D_e_ values. Fading rate tests showed that dimmer grains had higher fading rates, and Guo et al. [137] suggested that the discrepancy between the single-grain and single-aliquot D_e_ values were mainly due to the different fading rates of grains. However, a systematic increase in the fading-corrected D_e_ values with the ‘brightness threshold’ still existed and they proposed that brighter grains might still have slightly higher internal K contents than dimmer grains [137]. Guo et al. [137] have not observed a dependence of dose recovery ratios on the grain brightness. All the recovery ratios were close to unity irrespective of grain brightness, indicating that the sensitivity correction of natural signal (the first cycle in SAR protocol) is still successful for both bright and dim grains [137].

These studies presented above indicate that it is a ubiquitous phenomenon that brighter K-feldspar grains have higher D_e_ estimates, and can provide more reliable ages. By now, at least three factors may contribute to the lower D_e_ values for dim grains. One is that the dim grains have higher fading rates [129,137]. The second is that dim grains contain less internal K (less than 12–14 %), which corresponds to lower environmental dose rate [126,137]. The third is that sensitivity correction of natural signal is not successful for dim grains [108]. The true reason may be a combination of several factors and may also be sample-dependent. Therefore, in single-grain dating of K-feldspar, it is essential to exclude the dim grains with a ‘brightness threshold’, as age underestimation would be brought in if all grains are included to calculate the mean D_e_ value. A suitable ‘brightness threshold’ might be determined by the relationship between the mean D_e_ values and the ‘brightness threshold’—the ‘plateau’ method [108,137].

## 6. Conclusions

The pIRIR signals of K-feldspar can be sufficiently stable to provide accurate age estimations without fading correction. However, care should be taken to choose the suitable pIRIR signal to date samples with different ages. For young samples (e.g., <10 ka), the low-temperature signals (e.g., pIRIR_50, 180_, MET-pIRIR_170_) can be applied with a low preheat temperature (e.g., 200 °C) to avoid the age overestimation caused by residual doses. For intermediate-aged samples (e.g., 10–110 ka), the pIRIR_50, 290_, pIRIR_200, 290_, MET-pIRIR_250_ signals are all suitable. For older samples, with D_e_ larger than 400 Gy (~110 ka with a typical dose rate of ~3.5 Gy/ka), the pIRIR_50, 290_ would underestimate the D_e_ values, while the pIRIR_200, 290_, MET-pIRIR_250_ signals can still provide robust results. The empirical upper dating limit of 2D_0_ is also applicable to K-feldspar. With the modified protocols, such as ‘MAR with heat’ and ‘SAR with solar’, the dating limit can be increased to ~1500 Gy due to larger D_0_.

The SGC of K-feldspar can greatly save the machine time needed for D_e_ measurement, while it provides D_e_ estimates almost identical to those of the standard SAR procedure. In addition, with the SGC, a ‘mean L_n_/T_n_’ approach for D_e_ estimation can be performed for samples whose natural signals are close to the saturation level of the growth curve. In single-grain dating of K-feldspar, bright grains usually have higher D_e_ values than the dim grains. Suitable ‘brightness threshold’ should be applied to exclude the dim grains to avoid age underestimation.

## Figures and Tables

**Table 1:** Different pIRIR protocols: pIRIR_50, 225_; pIRIR_50, 180_; pIRIR_50, 290_; pIRIR_200, 290_; MET-pIRIR; ‘SAR with solar’; ‘MAR with heat’.

Step	pIRIR50,225	pIRIR50, 180	pIRIR50, 290	pIRIR200, 290	MET-pIRIR	‘SAR with Solar’	‘MAR with Heat’
	Buylaert et al. [37]	Reimann et al. [27]	Thiel et al. [38]	Li and Li [60]	Li and Li [26]	Li et al. [61]	Li et al. [62], Modified
1 *	Regenerative dose, D_i_	Regenerative dose, D_i_	Regenerative dose, D_i_	Regenerative dose, D_i_	Regenerative dose, D_i_	Regenerative dose, D_i_	Regenerative dose, D_i_
2	Preheat at 250 °C for 60 s	Preheat at 200 °C for 60 s	Preheat at 320 °C for 60 s	Preheat at 320 °C for 60 s	Preheat at 300 °C for 10 s	Preheat at 300 °C for 60 s	Preheat at 320 °C for 60 s
3	IR for 100 s at 50 °C	IR for 100 s at 50 °C	IR for 200 s at 50 °C	IR for 200 s at 200 °C	IR for 100 s at 50 °C	IR for 100 s at 50 °C	IR for 100 s at 50 °C
4	IR for 100 s at 225 °C	IR for 100 s at 180 °C	IR for 200 s at 290 °C	IR for 200 s at 290 °C	IR for 100 s at 100 °C	IR for 100 s at 100 °C	IR for 100 s at 100 °C
5	Test dose, D_t_	Test dose, D_t_	Test dose, D_t_	Test dose, D_t_	IR for 100 s at 150 °C	IR for 100 s at 150 °C	IR for 100 s at 150 °C
6	Preheat at 250 °C for 60 s	Preheat at 200 °C for 60 s	Preheat at 320 °C for 60 s	Preheat at 320 °C for 60 s	IR for 100 s at 200 °C	IR for 100 s at 200 °C	IR for 100 s at 200 °C
7	IR for 100 s at 50 °C	IR for 100 s at 50 °C	IR for 200 s at 50 °C	IR for 200 s at 200 °C	IR for 100 s at 250 °C	IR for 100 s at 250 °C	IR for 100 s at 250 °C
8	IR for 100 s at 225 °C	IR for 100 s at 180 °C	IR for 200 s at 290 °C	IR for 200 s at 290 °C	Test dose, D_t_	Test dose, D_t_	IR for 100 s at 300 °C
9	IR at 290 °C for 40 s	Return to step 1	IR at 325 °C for 100 s	IR at 325 °C for 100 s	Preheat at 300 °C for 10 s	Preheat at 300 °C for 60 s	Cutheat to 500 °C
10	Return to step 1		Return to step 1	Return to step 1	IR for 100 s at 50 °C	IR for 100 s at 50 °C	Test dose, D_t_
11					IR for 100 s at 100 °C	IR for 100 s at 100 °C	Preheat at 320 °C for 60 s
12					IR for 100 s at 150 °C	IR for 100 s at 150 °C	IR for 100 s at 50 °C
13					IR for 100 s at 200 °C	IR for 100 s at 200 °C	IR for 100 s at 100 °C
14					IR for 100 s at 250 °C	IR for 100 s at 250 °C	IR for 100 s at 150 °C
15					IR at 320 °C for 100 s	Solar simulator for 2 h	IR for 100 s at 200 °C
16					Return to step 1	Return to step 1	IR for 100 s at 250 °C
17							IR for 100 s at 300 °C

* For SAR protocols, in the first cycle, i = 0 and D_0_ = 0, and the natural signal is measured. The sequence is run with several regenerative doses including a zero dose and a repeat dose, to build the growth curve.

**Table 2:** Characteristic saturation dose (D_0_) of the growth curves from the conventional SAR protocol with different pIRIR signals.

Signal	D0 (Gy)	Test Dose (Gy)	Reference
pIRIR_50, 295_	204 ± 5	12	Qin and Zhou [79]
	286 ± 24	84	
pIRIR_50, 290_	203	4	Liu et al. [81]
	320	53	
	424	595	
pIRIR_50, 225_	159 ± 87	5	Colarossi et al. [80]
	455	60	
	435	120	
	526	200	
	556 ± 66	320	
pIRIR_50, 290_	290	32	Qin et al. [84]
	349	213	
pIRIR_50, 225_	367	32	
	433	213	
pIRIR_50, 170_	404	32	
	484	213	
MET-pIRIR_250_	327 ± 16	54	Li and Li [63]
MET-pIRIR_300_	250 ± 12	54	
MET-pIRIR_250_	324 ± 5	72.5	Zhang and Li [78]
	417 ± 9	145	
MET-pIRIR_300_	396 ± 13	300	Zhang et al. [85]
